# Early life phthalate exposure impacts gray matter and white matter volume in infants and young children

**DOI:** 10.1016/j.envres.2025.121826

**Published:** 2025-05-10

**Authors:** Emily J. Werder, Kun Lu, Chih-Wei Liu, Jake E. Thistle, Julia E. Rager, Gang Li, Zhengwang Wu, Tengfei Li, Li Wang, Dale P. Sandler, John H. Gilmore, Joseph Piven, Hongtu Zhu, Weili Lin, Stephanie M. Engel

**Affiliations:** a Department of Radiology, University of North Carolina at Chapel Hill, Chapel Hill, NC, United States; b Department of Epidemiology, University of North Carolina at Chapel Hill, Chapel Hill, NC, United States; c Epidemiology Branch, National Institute of Environmental Health Sciences, Research Triangle Park, NC, United States; d Department of Environmental Sciences and Engineering, University of North Carolina at Chapel Hill, Chapel Hill, NC, United States; e Biomedical Research Imaging Center, University of North Carolina at Chapel Hill, Chapel Hill, NC, United States; f Department of Psychiatry, University of North Carolina at Chapel Hill, Chapel Hill, NC, United States; g Department of Biostatistics, University of North Carolina at Chapel Hill, Chapel Hill, NC, United States

**Keywords:** Children’s environmental health, Endocrine disrupting chemicals, Neurodevelopment, Phthalates

## Abstract

**Objective:**

Prenatal phthalate exposure is associated with adverse neurodevelopmental outcomes, yet data on impacts of early life exposure remains limited. We investigated phthalate and replacement plasticizer exposures from 2 weeks to 7 years of age in relation to brain anatomical attributes, using serial structural magnetic resonance imaging (sMRI).

**Material and methods:**

Children were enrolled after birth into the UNC Baby Connectome Project, a longitudinal neuroimaging study (North Carolina, USA; 2017–2020). Urine samples (n = 406) were collected at each visit and analyzed for 17 phthalate and replacement plasticizer metabolites. Among 157 children contributing 369 sMRIs, we calculated metabolite-specific average exposures across each individual’s urine samples and used linear mixed models to estimate longitudinal associations of log transformed, specific gravity-adjusted average metabolite concentrations with gray and white matter volume, and cortical volume, thickness, and surface area. We examined sex-specific differences in these associations.

**Results:**

Higher average metabolite concentration was associated with lower gray matter volume (MCPP: (−1.73 cm^3^, 95 % CI: −3.36, −0.10) and higher white matter volume (∑DEHP: 2.28 cm^3^, 95 % CI: 0.08, 4.48). Among boys (n = 72, 140 sMRIs), MEP (−2.97 cm^3^, 95 % CI: −5.85, −0.09) and MiBP (−2.40 cm^3^, 95 % CI: −4.64, −0.15) were also associated with lower gray matter volume. Among females (n = 85, 229 MRIs), higher ∑DINCH exposure was associated with higher white matter volume (2.27 cm^3^, 95 % CI: 0.29, 4.25). We observed significant sex interactions for ∑DEHP with gray matter (p-interaction = 0.03) and ∑DINCH with white matter volume (p-interaction = 0.001).

**Conclusion:**

Early life phthalate/plasticizer exposure may differentially impact various brain region volumes in early childhood, with potential downstream consequences on functional development.

## 1. Introduction

The first years of life are a critical period of accelerated brain growth that lay the foundation for lifelong neurodevelopmental trajectories of cognition and behavior, as well as risk for neurodevelopmental disorders. This period of accelerated gray matter growth, rapid myelination of white matter, and maturation of structural and functional networks (Gilmore et al., 2018; Knickmeyer et al., 2008; Lebel and Deoni, 2018) coincides with the emergence of behavioral and cognitive abilities (Girault et al., 2019). Because brain development occurs via a precisely synchronized sequence of coordinated events, exposure to environmental toxicants at critical stages may disrupt essential processes, initiating a deleterious cascade that fundamentally alters neurobehavioral trajectories.

Children are exposed to a myriad of xenobiotic neurotoxicants (Landrigan and Goldman, 2011) in air, water, and dietary sources (Bennett et al., 2016; Gore et al., 2015; Schantz et al., 2020). The infant brain may be especially vulnerable to toxicants because fragile cerebral vessels in the neurovascular unit impact permeability of the blood brain barrier (Saunders et al., 2012), and immature toxicant metabolic systems constrain metabolism and excretion of toxicants (Cole et al., 2003; Costa et al., 2003). Infants no longer have the protection that was provided by the placenta during fetal development (Saunders et al., 2012), which may render them more vulnerable to toxicant exposures than the fetus. Infants and children are exposed to chemicals through hand-to-mouth behaviors, increased contact with the ground, breastfeeding, formula consumption, sustained use of baby products, and their diet.

Phthalates are a class of synthetic chemicals that are widely used as solvents and plasticizers in commercial and consumer products, such as medical devices, personal care products and cosmetics, food packaging, and building materials. Human exposure to phthalates is ubiquitous, occurring through dermal absorption, inhalation, and ingestion. Diet is a principal exposure pathway (Serrano et al., 2014). Due to mounting evidence that di(2-ethylhexyl) phthalate (DEHP) and dibutyl phthalate (DBP) are harmful to human health, their use has been restricted in children’s toys and other childcare products in the United States. Commonly used phthalate replacement plasticizers, including di (isononyl)cyclohexane-1,2-dicarboxylate (DINCH) and di-2-ethylhexyl terephthalate (DEHTP) (Wenzel et al., 2021; Rodriguez-Carmona et al., 2020), are increasingly found in population based sampling of the United States (Silva et al., 2013, 2017, 2019). In addition, we previously reported widespread detection of urinary metabolites of phthalates and replacements plasticizers in infancy and early childhood in the University of North Carolina (UNC) Baby Connectome Project (BCP) (Thistle et al., 2024). Although NHANES human biomonitoring data in the general US population includes only children ages 3 and older, available data indicate that younger age groups typically have higher concentrations of phthalate metabolites (Nguyen et al., 2019; Control CfD and Prevention, 2009). Indeed, in our own study, we observed age-related differences in exposure wherein phthalate levels were often highest in the first year of life and declined with age (Thistle et al., 2024).

Robust evidence demonstrates that prenatal phthalate exposure elicits adverse neurobehavioral, cognitive, and developmental impacts (Daniel et al., 2020; Ejaredar et al., 2015; Li et al., 2019; Lee et al., 2018), yet the structural and functional substrates underlying these relationships remain understudied. The few neuroimaging studies which have evaluated the role of phthalate exposure in neurodevelopment assess heterogeneous endpoints at varying ages (Wylie and Short, 2023). England-Mason et al. evaluated prenatal phthalates and white matter microstructure at 3–5 years of age in the Alberta Pregnancy Outcomes and Nutrition (APrON) study (n = 76) (England-Mason et al., 2020), reporting associations between high molecular weight phthalates and increased mean diffusivity of white matter tracts. In the Generation R study (n = 775), prenatal phthalates were associated with global brain volumes at age 10, with more prominent effects among females (Ghassabian et al., 2023). In a study of 49 teenagers, prenatal phthalates were associated with reduced regional brain volumes and altered white matter integrity at 13–16 (Shen et al., 2021). The only existing study of postnatal phthalate exposure and neuroimaging, a cross-sectional analysis of 8–11-year-olds diagnosed with attention deficit hyperactivity disorder (n = 115), reported that metabolites of di(2-ethylhexyl) phthalate (DEHP) were negatively correlated with cortical thickness in the right middle and superior temporal gyri (Park et al., 2014).

The nascent body of research investigating the links between phthalate exposure and neuroimaging biomarkers is largely restricted to prenatal exposures in relation to MRIs acquired later in adolescence. Studies linking prospective postnatal exposure, longitudinal scans, and imaging during sensitive periods of early development are needed to better understand the impacts of phthalate exposure on neurodevelopment during this critical window of development (Cajachagua-Torres et al., 2024). To address these gaps, we examined early life phthalate and replacement plasticizer exposure in relation to global measures of structural brain growth from two weeks to seven years of age in the UNC BCP. Based on previous evidence that neurodevelopmental effects of phthalate exposure are sexually dimorphic (Palanza et al., 2021; Radke et al., 2020), we additionally evaluated differences in associations between early life phthalate exposure and global brain measures by child sex.

## 2. Methods

### 2.1. Study population

The BCP is a longitudinal study that aims to characterize normative brain and behavioral development in typically developing infants across the first 5 years of life (Howell et al., 2019). Eligible participants were born between 37 and 42 weeks’ gestation, with birth weight appropriate for gestational age, absence of pregnancy complications, and parents reported no medical or genetic conditions related to neurodevelopment. The BCP enrolled participants between birth and age five at two sites, the University of Minnesota and the University of North Carolina – Chapel Hill (UNC), between 2017 and 2020. To limit participant attrition, the BCP employed an accelerated cohort design, with infants and children entering observation in a staggered pattern and followed forward with longitudinal scans spanning different periods of early life. This dense longitudinal sampling within developmental periods maximizes coverage across a wider age range while relying on a shorter follow-up period (Supplemental Fig. 1).

The UNC BCP site (n = 238) additionally attempted to collect a urine sample at every participant visit. The present study is limited to UNC BCP participants who provided at least one urine sample (n = 187) for exposure ascertainment, completed at least one MRI (n = 159), and had known covariate information, for an analytic sample of 157 participants contributing 406 urines and 369 scans.

Parents of all participants provide permission and informed consent prior to participation and the study was approved by the University of North Carolina at Chapel Hill Institutional Review Board.

### 2.2. Urine sample collection

Urine samples were collected at each attempted imaging appointment. For infants and young children wearing diapers, research assistants provided parents with a fresh diaper (Bambo Nature Premium) and Waterwipes to remove residual lotion or creams prior to placing 4–5 hospital-grade cotton balls in the diaper at the beginning of the visit. After the visit, cotton balls were collected in a sterile, phthalate-free container and stored at 4 °C. For toilet trained children, a sterile phthalate-free toilet hat was placed in the toilet to catch urine at any time during the visit. Once collected, samples were sent to the UNC BioSpecimen Processing Facility (median processing time ~15 h). Urine was extracted from cotton balls using a phthalate-free syringe and stored securely. Samples were aliquoted and stored at −80 °C within 24 h of processing.

### 2.3. Phthalate exposure measurement

The detailed protocol for urine sample preparation and mass spectrometry analysis have been described previously (Hammel et al., 2023). Briefly, concentrations were quantified using highly sensitive triple quadrupole mass spectrometry with ultra-high performance liquid chromatography for 17 metabolites of phthalates and replacement plasticizers: monobutyl phthalate (MnBP), mono-3carboxypropyl phthalate (MCPP), monoisobutyl phthalate (MiBP), monoethyl phthalate (MEP), monobenzyl phthalate (MBzP), mono-2ethylhexyl phthalate (MEHP), mono-2-ethyl-5-hydroxyhexyl phthalate (MEHHP), mono-2-ethyl-5-oxohexyl phthalate (MEOHP), mono-2-ethyl5-carb oxypentyl phthalate (MECPP), mono-2-carboxymethylhexyl phthalate (MCMHP), monooxononyl phthalate (MONP), monoisononyl phthalate (MCOP), monocarboxyisononyl phthalate (MCNP), cyclohexane-1, 2-dicarboxylic acid, monohydroxy isononyl ester (MHNCH), cyclohexane-1,2-dicarboxylic acid, monocarboxy isooctyl ester (MCOCH), mono-2-ethyl-5-hydrohexyl terephthalate (MEHHTP), and mono-2-ethyl-5-carboxypentyl terephthalate (MECPTP). All metabolite concentrations were adjusted for specific gravity (SG) (Thistle et al., 2024), an appropriate approach to account for urinary dilution in infants and young children (Pearson et al., 2009; Wang et al., 2015). Concentrations below the limit of detection (LOD) were imputed using the LOD/√2. We calculated molar sums (μmol/L) of metabolite concentrations by summing SG-adjusted and LOD/√2-imputed metabolite concentrations after dividing by the molecular weight, for three compounds: DEHP (∑DEHP = MEHP + MECPP + MEHHP + MEOHP + MCMHP); DINCH (∑DINCH = MCOCH + MHNCH); and DEHTP (∑DEHTP = MECPTP + MEHHTP). Association analyses were completed for eight distinct exposures based on 14 metabolites: five individual metabolite exposures (MnBP, MCPP, MiBP, MEP, MBzP), and 3 M sums (DEHP, DINCH, DEHTP) derived from nine individual metabolites. The remaining three metabolites we measured (MONP, MCOP, MCNP) were omitted from statistical analyses because they were detected in less than 10 % of samples.

Three metabolites (MONP, MCOP, MCNP) were omitted from statistical analyses because they were detected in less than 10 % of samples.

### 2.4. Neuroimaging acquisition and processing

The BCP imaging protocol (Howell et al., 2019), which was based on the imaging protocol developed by the Human Connectome Project (Van Essen et al., 2013), is validated and specified for acquiring high quality infant brain MRIs. The protocol consists of sMRI T_1_-weighted (T_1_w) and T_2_-weighted (T_2_w), resting-state functional MRI (rsfMRI), and diffusion MRI (dMRI). Images were acquired on 3T Siemens Prisma MRI scanners using a Siemens 32 channel head coil at the UNC Biomedical Research Imaging Center (BRIC). If considerable motion was observed at the time of scanning, study personnel attempted reacquisition if possible. To ensure image processing quality, we conducted quality control in two stages. Before processing, raw MR images were visually checked for motion artifacts, including excessive motion, insufficient coverage, and ghosting. Any images with severe motion artifacts were excluded. Structural MR images were then processed using the iBEAT V2.0 pipeline (http://www.ibeat.cloud) (Wang et al., 2023). After processing, an experienced infant MR imaging processing expert inspected the processing results to ensure that tissue segmentation and cortical surfaces were correctly reconstructed. The sMRI T_1_w and T_2_w images provide information regarding structural brain development and global morphology. Outcomes of interest included global volumes (cm^3^): gray matter (GMV), white matter (WMV), and cortical gray matter (CV); cortical thickness (CT, mm), and cortical surface area (CSA, cm^2^). We used intracranial volume (cm^3^) as an adjustment factor for scanning effects.

### 2.5. Statistical analysis

For phthalate and replacement plasticizer exposures, we log transformed concentrations and then averaged across all samples provided by an individual. Thus, we assigned each participant eight exposure metrics, each corresponding to the average concentration of a specific metabolite or molar sum. We additionally calculated cumulative average exposures for each exposure metabolite (n = 5) or molar sum (n = 3) at each MRI, averaging concentrations from all of an individual’s samples until the date of the index MRI (but excluding those provided after the scan date). Because phthalates are rapidly metabolized and have short half-lives (Johns et al., 2015), we prioritized the *total* average concentrations (as opposed to *cumulative* average concentrations) to leverage all available exposure information, better represent typical early life exposure, and maximize sample size and statistical power in primary analyses. Cumulative average exposures were analyzed in sensitivity analyses (n = 284).

To account for repeated scans, and the correlation introduced with multiple observations per participant, we used linear mixed models with temporal power covariance to estimate longitudinal associations between average phthalate/plasticizer exposure and each of the global sMRI region outcomes. All models were adjusted for total intracranial volume, child’s sex assigned at birth, maternal age at delivery (continuous years), and child’s age on the date of the MRI (days). Due to the strong age-dependence of brain growth during early childhood, we adjusted for child’s age using natural cubic splines with knots at the 10th (115 days), 25th (335 days), 75th (1198 days), and 90th (1713 days) percentile. For white matter volume, we included an additional knot at the 5th percentile (61 days) to improve model fit for this outcome. Knot selection and model specification were guided by goodness-of-fit tests and graphical interpretation of residuals. We modeled each exposure-outcome combination separately, reporting effect estimates as the amount of change in MRI feature (β coefficient) associated with a log-unit increase in exposure, and corresponding 95 % confidence intervals (CIs). To account for multiple hypothesis testing, we calculated false discovery rate (FDR) corrected p-values for 40 tests (8 exposures and 5 outcomes) (Benjamini and Hochberg, 1995).

Because phthalates are endocrine disrupting chemicals (Engel and Wolff, 2013) and early life brain growth trajectories are sex-dependent (Gilmore et al., 2007), we estimated sex-specific associations in stratified models restricted to males (n = 72 participants, 140 observations) or females (n = 85 participants, 229 observations) with the same covariate adjustment set as the overall analysis. We evaluated sex differences in the overall sample using the augmented product term approach (Buckley et al., 2017), reporting the p-value associated with the two-sided Wald test statistic (alpha = 0.05) for the product term between exposure and sex.

Sensitivity analyses evaluated time-varying cumulative average exposures and exposure-age interactions dichotomized at 12-,18-, and 24-months. All statistical analyses were conducted in SAS version 9.4 (Cary, NC, USA).

## 3. Results

The sex distribution in the study sample was approximately balanced (54 % female), though females contributed slightly more observations (n = 229, 62 % of scans) (Table 1). The youngest and oldest ages at MRI were at 6 days and 2567 days (~7 years old), respectively. The majority of scans (61 %) were obtained before age 2 years (Table 1). Participants contributed a median of 2 MRIs (maximum = 8) and 2 urine samples (maximum = 7). Detection rates for individual metabolites ranged from 83 % (MEP) to 100 % (MBzP, MnBP), and from 66 % (DINCH) to 100 % for molar sums (data not shown).

In the overall sample, MCPP was associated with lower GMV (−1.7 cm^3^, 95 % CI: −3.4, −0.1), whereas ∑DEHP was associated with higher WMV (2.3 cm^3^, 95 % CI: 0.1, 4.5) (Table 2). Although not statistically significant, MnBP was suggestively associated with lower GMV (−2.0 cm^3^, 95 % CI: −4.1, 0.05). We did not observe any significant associations with CV (Table 3), though patterns were similar between GMV and CV. Exposure associations with CV were generally slightly attenuated and less precise compared to corresponding estimates for GMV. MiBP was associated with lower cortical thickness (−0.01 mm, 95 % CI: −0.02, −0.004). While no exposures were significantly associated with cortical surface area, ∑DEHP was associated with a 10.4 cm^2^ increase in CSA (95 % CI: −0.02, 20.9).

**Fig. 1.**
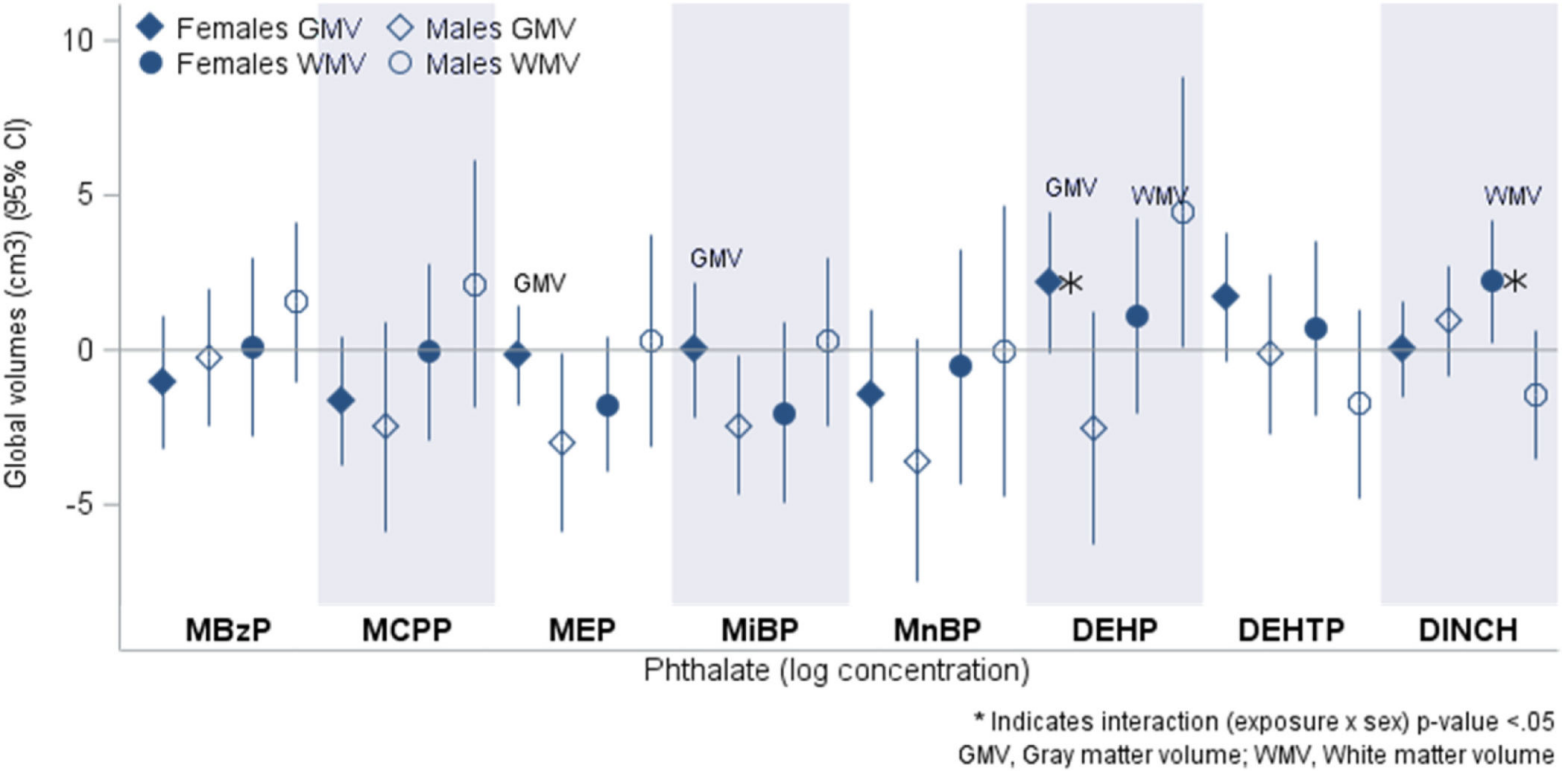
Associations of total average phthalate exposure and global brain volumes by sex in the UNC BCP (n = 229 females and 140 males).

When considering sex-specific associations, main effects appeared to be driven by males (Fig. 1). Among males, we observed inverse associations with GMV (MEP: −3.0 cm^3^, 95 % CI: −5.9, −0.1; MiBP: −2.4 cm^3^, 95 % CI: −4.6, −0.2) and a positive association between ∑DEHP and WMV (4.5 cm^3^, 95 % CI: 0.1, 8.9) (Table 2). Although there was no main effect between ∑DINCH and WMV in the overall sample, among females ∑DINCH was associated with a 2.3 cm^3^ increase in WMV (95 % CI: 0.3, 4.2, interaction p-value = 0.001). We also observed heterogeneity by sex in the relationship between ∑DEHP and GMV (interaction p-value = 0.03), with a suggestive positive association among females (2.2 cm^3^, 95 % CI: −0.05, 4.5) and a nonsignificant inverse association among males (−2.5 cm^3^, 95 % CI: −6.3, 1.2). There were no statistically significant sex differences for the cortical measures (Table 3), though differences were suggestive for MBzP with CSA (interaction p-value = 0.07) and ∑DEHP with CT (interaction p-value = 0.09). Further, the main effect with MiBP and lower cortical thickness was also observed when restricted to males only (−0.02 mm, 95 % CI: −0.03, −0.004).

In sensitivity analyses of cumulative average exposure, patterns of associations were similar to those reported in main analyses using total average exposure, albeit most were slightly attenuated (Supplemental Tables 1–2, Supplemental Fig. 2). When we attempted to assess critical windows of exposure by dichotomizing the sample at ages 12- and 18-months, analyses were underpowered and models encountered convergence problems. As such, we were unable to interpret these analyses (data not shown). In age-stratified analyses dichotomized at two years old (Supplemental Table 3), we did not observe evidence of a statistical interaction between phthalate/replacement plasticizer exposure and age (divided at two years). Main effects that we observed with GMV in the overall sample were observed in the younger age group only (MCPP: −2.7 cm^3^, 95 % CI: −4.7, −0.6; MiBP: −2.3 cm^3^, 95 % CI: −4.1, −0.4). Conversely, the positive association between DEHP and WMV in the main sample appeared to be driven by children older than two years (3.9 cm^3^, 95 % CI: 0.5, 7.4). When we corrected p-values for multiple testing, no associations passed the FDR threshold (data not shown).

## 4. Discussion

We found that higher early life urinary concentration of MCPP was associated with lower GMV, and ∑DEHP was associated with higher WMV, in children two weeks to seven years old. Relationships between phthalates and global brain morphology were more apparent among males. There was a general pattern of inverse associations between phthalate metabolite concentrations and GMV among males (e.g., MCPP, MEP, MiBP, and MnBP), though some associations were not statistically significant. Notably, the replacement plasticizer DINCH was associated with higher WMV among females only. With the exception of MiBP and slightly lower cortical thickness, we did not observe effects of phthalate/plasticizer exposure on cortical measures. In age-stratified analyses, we observed associations with lower GMV (e.g., MCPP and MiBP) in children under age two, and associations between DEHP and higher WMV in children older than age two. No associations remained significant when we corrected p-values for multiple hypothesis testing (n = 40 tests). Because selection of exposures and outcomes was hypothesis-driven, we prioritized results that were not FDR-adjusted.

The present study is the first to examine early life phthalate exposures in association with brain MRI in a longitudinal setting. Strengths of our approach include repeated biomarkers of exposure across the study period, repeated neuroimaging with dense longitudinal sampling during sensitive periods of development, sufficient sample size to evaluate sexually dimorphic associations, and inclusion of the emerging replacement plasticizers DINCH and DEHTP. We note that our study also has important limitations. We elected to use average phthalate concentrations to better represent typical exposure across the study period because phthalates are non-persistent chemicals with short half-lives. Intraclass correlations for phthalate metabolites and replacement plasticizers among participants with multiple samples in the UNC BCP were previously reported (range, ρ = 0.10–0.48), indicating low to moderate reliability and high within-individual variation (Thistle et al., 2024). In sensitivity analyses, we evaluated phthalates as average cumulative exposures until each MRI scan date to determine whether exposure information obtained after the index MRI was influencing results. Interpretation of these analyses were similar to those estimated using total average exposures, suggesting that exposure measurement error did not influence results. To maximize power, we presented total average exposures as primary analyses (n_total_ = 369 versus n_cumulative_ = 284), with cumulative average exposures as supplemental information. While age-specific analyses were underpowered, previous analyses of phthalate and plasticizer exposure levels in this population revealed that concentrations of MnBP, MCPP, MiBP, MEP, and MBzP were inversely associated with age, and often highest before age one (Thistle et al., 2024). Based on this pattern, and the age-dependence of brain growth during early life, we hypothesized that critical windows may exist in the relationship between phthalates and brain morphology. However, the temporal distribution of data undermined our ability to evaluate exposure-age interactions because age-stratified sample sizes were prohibitively small. Our approach implementing separate single chemical models for each exposure did not address potential confounding by co-exposures, though correlations among individual metabolites and molar sums were modest (ranging from 0.23 to 0.52). A mixture approach that more accurately models combined real-world exposures, estimates the combined mixture effect, or identifies leading constituents, could be informative in future analyses. Finally, owing to study protocol constraints, most UNC BCP participants lived in close proximity to UNC and the study population over-represents white participants of higher socioeconomic and educational backgrounds. This lack of diversity limits the generalizability of our results to more diverse populations, particularly in the context of racial/ethnic and socioeconomic disparities in phthalate exposure (Welch et al., 2023) and socioeconomic impacts on early life brain growth (Triplett et al., 2022; Rakesh and Whittle, 2021). It is possible that associations may have been more stark in populations with higher exposure and fewer community and family resources capable of mitigating harms from environmental toxicants.

Although no other studies have assessed postnatal phthalate exposure with neuroimaging at the early ages we evaluated, one study evaluated prenatal maternal urinary phthalates in association with global brain volumes at age 10 in the Generation R cohort (n = 775) (Ghassabian et al., 2023). The authors reported inverse relationships between prenatal concentrations of MiBP (β = −8.11, 95 % CI: −13.56, −2.66) and MEP (β = −10.7, 95 %CI: −18.12, −3.28) with GMV. In our analysis, we also observed inverse associations for these metabolites and GMV, however these associations were observed among males only in our sample. Interestingly, sex-specific effects were observed predominantly among females in Generation R, whereas sex-specific patterns were more apparent among males in our analyses. Discrepancies between sex-specific associations in our study and Generation R may be related to sex differences in timing of exposure (prenatal versus postnatal) or neurodevelopment (infancy and childhood versus adolescence). We did observe higher WMV in association with DINCH exposure among females in our study, but the Generation R study didn’t measure DINCH, so we couldn’t compare this relationship.

Because DINCH is a replacement plasticizer that was introduced in 2002 as an alternative to DEHP and other high molecular weight phthalates (Kasper-Sonnenberg et al., 2019), it has not yet been widely studied. We identified one study, the Exposure of Children and Adolescents to Selected Chemicals Through Their Habitat Environment in Slovenia (CRP-SLO), that reported cross-sectional inverse associations between DINCH and serum brain-derived neurotrophic factor (BDNF) among 6–11-year-old boys (but not girls), though they did not observe a direct relationship between DINCH and neurobehavioral outcomes (Salamanca-Fernandez et al., 2024). The authors caution that meaningful interpretation of serum BDNF as a biomarker of neurodevelopmental effect remains elusive.

Many studies linking phthalate exposures with altered neurodevelopment, including externalizing behaviors and attention-deficit hyperactivity disorder (ADHD), find that males are often at higher risk for poorer neurodevelopmental outcomes (Ejaredar et al., 2015). Though the endocrine-disrupting properties of phthalates are well known (Cowell and Wright, 2017), modification of associations with neurodevelopment by sex is variable across studies. These inconsistencies may be due to heterogeneity in neurodevelopmental endpoints and assessments. In the present study, we observed sex-specific effects, linking multiple phthalate metabolite exposures (e. g., MEP, MiBP, and DEHP) to reduced GMV among males. Taken together with prior work reporting associations between prenatal MiBP and adverse executive function and behavioral problems in preschool-aged (Choi et al., 2021) and 6–10 year old males (Kobrosly et al., 2014), these results suggest that sex-specific alterations in gray matter growth could possibly underlie relationships between MiBP and neurobehavior.

A robust literature links regional and global gray matter deficits to inattention, hyperactivity, impulsivity, and ADHD. Vetter et al. report lower GMV in males with ADHD and oppositional defiance disorder compared to typically developing males aged 11–17 years old (Vetter et al., 2019). A study in the Adolescent Brain Cognitive Development Study (n = 10,692) found that inattentive, hyperactivity, and impulsivity traits show GMV deficits in areas associated with attention and emotion regulation, visuospatial processing and memory, and motor activity at 9–10 years of age (Reimann et al., 2024). A 2024 meta-analysis found that pediatric bipolar disorder and ADHD share decreased regional GMV in areas related to emotion processing and attention (Long et al., 2024). We offer evidence that the neural substrates connecting phthalate exposures and neurobehavioral outcomes could involve disruptions in gray matter development. Moreover, endocrine disrupting properties seem to be eliciting stronger effects on these neural substrates, and their subsequent neurobehavioral endpoints, in males.

The neurotoxicity of DEHP has been studied extensively, with frequently reported sex differences in associations with neurobehavior and autism spectrum disorders (Liu et al., 2023). We observed that DEHP concentrations were associated with higher WMV overall, and lower GMV among males. In the APrON study (n = 448), higher exposure to maternal prenatal DEHP was associated with poorer motor and executive function at two years old, with worse outcomes in males (Dewey et al., 2023). A subset of this study (n = 76) acquired neuroimages at 3–5 years of age and found that phthalate exposures were associated with impaired white matter microstructure integrity (England-Mason et al., 2020). In a study of six-year-olds in Korea, childhood DEHP exposure had an adverse effect on IQ and attentional performance (n = 175) (Kim et al., 2017). A cross-sectional analysis of phthalates and MRI at ages 6–15 (n = 115) found that DEHP metabolites were: negatively correlated with cortical thickness in some regions, significantly higher in males with ADHD than in males without ADHD, and the metabolite concentration was positively correlated with the severity of externalizing symptoms (Park et al., 2014). Despite being regulated in childhood toys and childcare products, we observed that children continue to be exposed to DEHP, likely due to diet being a major source of exposure (U.S. Consumer Product Safety Commission, 2014). Moreover, this early life exposure to DEHP impacts structural brain growth with potential implications for future neurodevelopment across early childhood and into adolescence.

In summary, our findings demonstrate that early life phthalate exposure is associated with structural brain growth in the first years of life, particularly among males. The associations and sex differences we note here contribute to understanding the neural basis through which phthalates may disrupt emotional, behavioral, and cognitive development. The observed association between DINCH and WMV suggests that the safety of replacement plasticizers should be carefully considered. Future studies should assess regional and subcortical measures, functional connectivity, and neurobehavioral outcomes.

## Supplementary Material

MMC1

Appendix A. Supplementary data

Supplementary data to this article can be found online at https://doi.org/10.1016/j.envres.2025.121826.

GMV, gray matter volume (cm^3^); WMV, white matter volume (cm^3^); CI, confidence interval; MBzP, monobenzyl phthalate; MCPP, mono-3carboxypropyl phthalate; MEP, monoethyl phthalate; MiBP, monoisobutyl phthalate; MnBP, monobutyl phthalate; DEHP, di(2-ethylhexyl) phthalate; DEHTP, di-2-ethylhexyl terephthalate; DINCH, di(isononyl) cyclohexane-1,2-dicarboxylate; effect estimates are per log-unit increase in SG-adjusted average urinary phthalate concentrations (log(ng/mL) for metabolites and log(μmol/L) for molar sums); models are stratified by child’s sex and adjusted for maternal age at delivery, child’s age (days) on the date of the MRI, and intracranial volume; interaction p-value is for product term between phthalate exposure and child’s sex in augmented product term model.

## Figures and Tables

**Table 1:** UNC Baby Connectome Project cohort.

Participant demographics (n = 157)	

Female, N(%)	85 (54)
Male, N(%)	72 (46)
Maternal age (years), median (IQR)	31.8 (5.0)
Number of urines, median (range)	2.0 (6.0)
Number of MRI scans, median (range)	2.0 (7.0)
Average phthalate exposures (n = 406)	Median (IQR)

MBzP, log(ng/mL)	1.7 (1.1)
MCPP, log(ng/mL)	0.8 (0.9)
MEP, log(ng/mL)	2.4 (1.1)
MiBP, log(ng/mL)	2.5 (1.1)
MnBP, log(ng/mL)	2.7 (0.8)
∑DEHP, log(μmol/L)	−2.6 (0.9)
∑DEHTP, log(μmol/L)	−1.5 (1.0)
∑DINCH, log(μmol/L)	−5.6 (1.4)
MRIs (n = 369)	Median (IQR)

Cortical volume, cm^3^	387.9 (106.1)
Gray matter volume, cm^3^	472.7 (123.9)
Intracranial volume, cm^3^	1112.0 (302.9)
White matter volume, cm^3^	334.4 (113.5)
Cortical thickness, mm	2.1 (0.1)
Cortical surface area, cm^2^	1817.4 (427.5)
Age at MRI	N (%)

≤1 year	106 (29)
>1–2 years	119 (32)
>2–4 years	78 (21)
>4–7 years	66 (18)

Female, number of MRI scans	229 (62)
Male, number of MRI scans	140 (38)

IQR, interquartile range; MBzP, monobenzyl phthalate; MCPP, mono-3carboxypropyl phthalate; MEP, monoethyl phthalate; MiBP, monoisobutyl phthalate; MnBP, monobutyl phthalate; DEHP, di(2-ethylhexyl) phthalate; DEHTP, di-2-ethylhexyl terephthalate; DINCH, di(isononyl)cyclohexane-1,2-dicarboxylate; MRI, magnetic resonance imaging.

Phthalate metabolite concentrations: log(ng/mL); molar sum concentrations: log(μmol/L); concentrations are adjusted for specific gravity and concentrations below the limit of detection (LOD) were imputed using the LOD/√2.

MRI volumes (cortical, gray matter, intracranial, white matter): cm^3^; cortical thickness: mm; cortical surface area: cm^2^.

**Table 2:** Associations of total average phthalate exposures with global gray and white matter volumes in the UNC BCP (n = 157 participants, n = 369 scans).

Exposure	Brain volume	Overall (n = 157)	Females (n = 85)	Males (n = 72)	Interaction p-value
		(n = 369 scans)	(n = 229 scans)	(n = 140 scans)	
		β (95 % CI)	β (95 % CI)	β (95 % CI)	
MBzP	GMV	−0.5 (−1.9, 0.9)	−1.0 (−3.2, 1.1)	−0.2 (−2.4, 2.0)	0.7
	WMV	1.0 (−0.7, 2.8)	0.1 (−2.7, 3.0)	1.6 (−1.0, 4.2)	0.3
MCPP	GMV	**−1.7 (−3.4, −0.1)**	−1.6 (−3.7, 0.5)	−2.4 (−5.8, 0.9)	0.5
	WMV	0.8 (−1.3, 2.8)	−0.04 (−2.9, 2.8)	2.2 (−1.8, 6.2)	0.2
MEP	GMV	−0.6 (−1.9, 0.7)	−0.2 (−1.8, 1.5)	**−3.0 (−5.9, −0.1)**	0.07
	WMV	−0.2 (−1.8, 1.4)	−1.8 (−3.9, 0.4)	0.3 (−3.1, 3.7)	0.3
MiBP	GMV	−1.2 (−2.6, 0.2)	0.03 (−2.2, 2.2)	**−2.4 (−4.6, −0.2)**	0.07
	WMV	−0.7 (−2.5, 1.0)	−2.0 (−4.9, 0.9)	0.3 (−2.4, 3.0)	0.2
MnBP	GMV	−2.0 (−4.1, 0.05)	−1.5 (−4.3, 1.4)	−3.6 (−7.5, 0.4)	0.2
	WMV	−0.2 (−2.8, 2.3)	−0.5 (−4.3, 3.3)	−0.03 (−4.7, 4.7)	0.6
∑DEHP	GMV	0.9 (−1.0, 2.7)	2.2 (−0.05, 4.5)	−2.5 (−6.3, 1.2)	**0.03**
	WMV	**2.3 (0.1, 4.5)**	1.1 (−2.0, 4.3)	**4.5 (0.1, 8.9)**	0.4
∑DEHTP	GMV	0.8 (−0.7,2.2)	1.7 (−0.4, 3.9)	−0.1 (−2.7,2.5)	0.3
	WMV	−0.4 (−2.2, 1.4)	0.7 (−2.1, 3.6)	−1.7 (−4.8, 1.4)	0.2
∑DINCH	GMV	0.4 (−0.7, 1.4)	0.1 (−1.5, 1.6)	1.0 (−0.8, 2.8)	0.5
	WMV	0.7 (−0.6, 2.0)	**2.3 (0.3, 4.3)**	−1.4 (−3.5, 0.6)	**0.001**

GMV, gray matter volume (cm^3^); WMV, white matter volume (cm^3^); CI, confidence interval; MBzP, monobenzyl phthalate; MCPP, mono-3carboxypropyl phthalate; MEP, monoethyl phthalate; MiBP, monoisobutyl phthalate; MnBP, monobutyl phthalate; DEHP, di(2-ethylhexyl) phthalate; DEHTP, di-2-ethylhexyl terephthalate; DINCH, di(isononyl)cyclohexane-1,2-dicarboxylate; effect estimates (β coefficients) are per log-unit increase in SG-adjusted average urinary phthalate concentrations (log(ng/mL) for metabolites and log (μmol/L) for molar sums); models are adjusted for maternal age at delivery, child’s sex, child’s age (days) on the date of the MRI, and intracranial volume; sex-specific estimates are estimated from stratified models; interaction p-value is for product term between total average phthalate exposure and child’s sex; boldface type indicates association with p-value <0.05.

**Table 3:** Associations of total average phthalate exposures with cortical surface area, thickness, and volume in the UNC BCP (n = 369).

Phthalate	Cortical measure	Overall (n = 369)	Females (n = 229)	Males (n = 140)	Interaction p-value
		β (95 % CI)	β (95 % CI)	β (95 % CI)	
MBzP	CSA	−0.03 (−8.2, 8.1)	−10.5 (−22.2, 1.3)	8.6 (−3.5, 20.7)	0.07
	CT	−0.001 (−0.01, 0.01)	0.01 (−0.004, 0.02)	−0.01 (−0.02, 0.005)	0.3
	CV	−0.2 (−1.4, 1.0)	−1.4 (−3.3, 0.5)	0.5 (−1.5, 2.4)	0.4
MCPP	CSA	−1.0 (−10.5, 8.4)	−1.7 (−12.8, 9.5)	0.8 (−17.8, 19.5)	1.0
	CT	−0.002 (−0.01, 0.01)	0.000 (−0.01, 0.01)	−0.01 (−0.02, 0.01)	0.8
	CV	−1.2 (−2.6, 0.2)	−1.4 (−3.4, 0.5)	−1.3 (−4.3, 0.7)	0.7
MEP	CSA	−4.1 (−11.5,3.3)	−6.6 (−15.0, 1.8)	−0.2 (−16.2, 15.8)	0.9
	CT	0.002 (−0.01, 0.01)	0.01 (−0.003, 0.02)	−0.01 (−0.02, 0.01)	0.7
	CV	−0.7 (−1.8, 0.4)	−1.1 (−2.6, 0.4)	−1.8 (−4.3, 0.7)	0.2
MiBP	CSA	7.4 (−0.9, 15.6)	−9.8 (−1.7, 21.2)	7.2 (−5.4, 19.7)	0.6
	CT	**−0.01 (−0.02, −0.004)**	−0.01 (−0.02, 0.003)	**−0.02 (−0.03, −0.004)**	0.8
	CV	−0.4 (−1.6, 0.8)	0.01 (−2.0, 2.0)	−1.0 (−3.0, 1.0)	0.2
MnBP	CSA	−1.3 (−13.1, 10.4)	−0.8 (−15.8, 14.20	−1.8 (−23.6, 20.0)	0.7
	CT	−0.01 (−0.02, 0.003)	−0.004 (−0.02, 0.01)	−0.02 (−0.04, 0.01)	0.8
	CV	−1.7 (−3.4, 0.1)	−1.5 (−4.1, 1.1)	−2.7 (−6.1, 0.7)	0.2
∑DEHP	CSA	10.4 (−0.2, 20.9)	10.6 (−1.7, 22.8)	8.7 (−12.1, 29.5)	0.9
	CT	−0.01 (−0.02, 0.01)	−0.001 (−0.01, 0.01)	−0.02 (−0.04, 0.004)	0.09
	CV	0.7 (−0.9, 2.2)	1.2 (−1.0, 3.3)	−1.0 (−4.2, 2.3)	0.3
∑DEHTP	CSA	6.4 (−2.1, 14.9)	9.7 (−1.5, 20.8)	2.3 (−11.9, 16.5)	0.4
	CT	−0.01 (−0.01, 0.003)	−0.004 (−0.02, 0.01)	−0.01 (−0.02, 0.01)	0.8
	CV	0.8 (−0.4, 2.1)	1.6 (−0.3, 3.5)	0.1 (−2.1, 2.4)	0.4
∑DINCH	CSA	0.6 (−5.4, 6.7)	1.0 (−7.3, 9.3)	1.5 (−8.3, 11.3)	0.6
	CT	0.002 (−0.01, 0.01)	0.003 (−0.01, 0.01)	0.001 (−0.01, 0.01)	0.3
	CV	0.3 (−0.6, 1.2)	0.1 (−1.3, 1.5)	0.6 (−0.9, 2.1)	0.5

CSA, cortical surface area (cm^2^); CT, cortical thickness (mm); CV, cortical volume (cm^3^); CI, confidence interval; MBzP, monobenzyl phthalate; MCPP, mono-3carboxypropyl phthalate; MEP, monoethyl phthalate; MiBP, monoisobutyl phthalate; MnBP, monobutyl phthalate; DEHP, di(2-ethylhexyl) phthalate; DEHTP, di-2-ethylhexyl terephthalate; DINCH, di(isononyl)cyclohexane-1,2-dicarboxylate; effect estimates (β coefficients) are per log-unit increase in SG-adjusted average urinary phthalate concentrations (log(ng/mL) for metabolites and log(μmol/L) for molar sums); models are adjusted for maternal age at delivery, child’s sex, child’s age (days) on the date of the MRI, and intracranial volume; sex-specific estimates are estimated from stratified models; interaction p-value is for product term between total average phthalate exposure and child’s sex; boldface type indicates association with p-value <0.05.

## Data Availability

Data will be made available on request.
