# Supplementary material for: Early life phthalate exposure impacts gray matter and white matter volume in infants and young children
Source: Environ Res. Author manuscript; Available in PMC 2025 Sep 22. (PMC12452038; doi:10.1016/j.envres.2025.121826)
Supplement: MMC1 [file NIHMS2089351-supplement-MMC1.docx]

**Supplemental Tables & Figures**


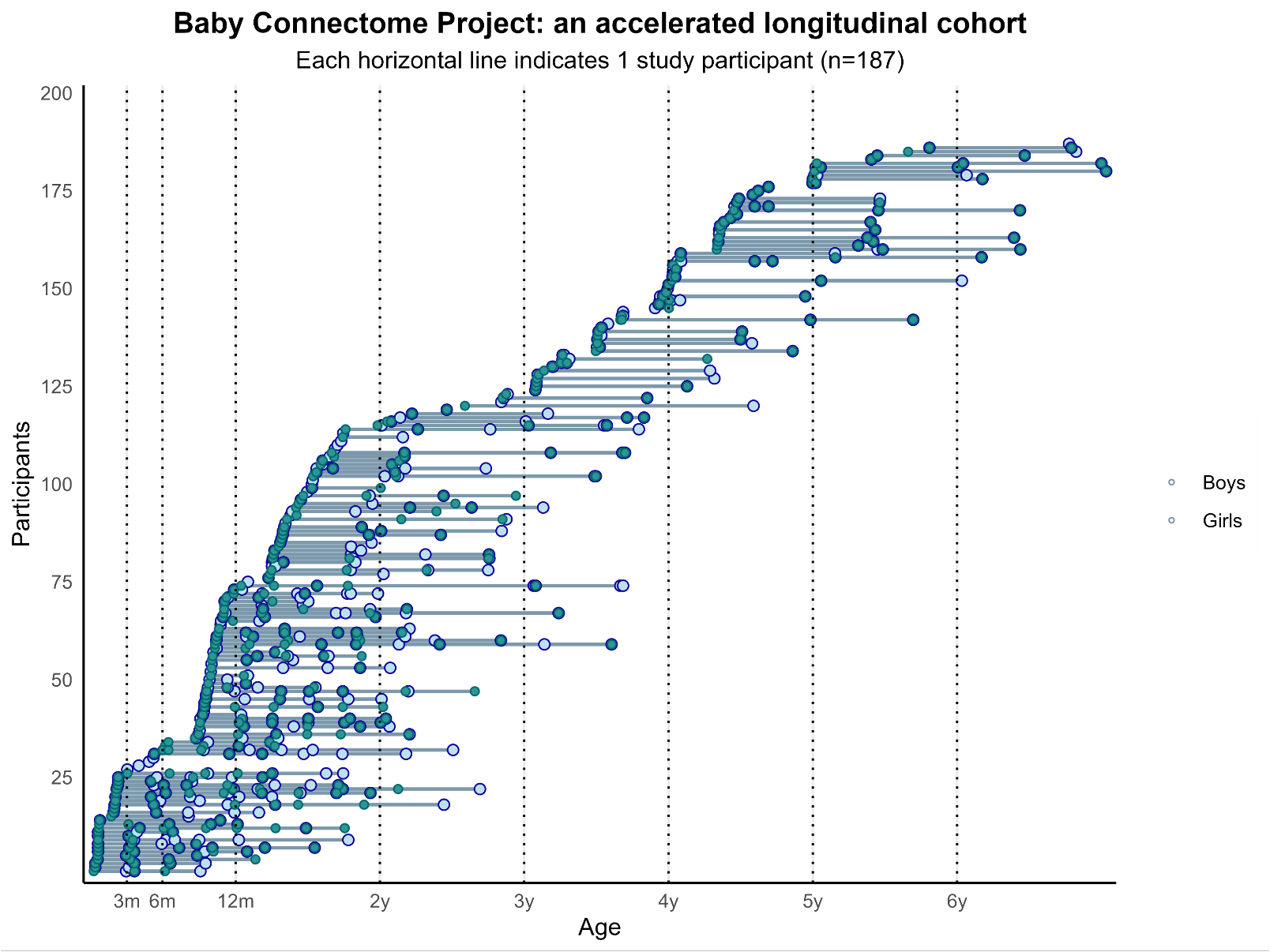


Supplemental Figure 1. Enrollment in the UNC BCP from 2017-2020. Blue circles represent urine samples, green circles represent MRIs, and green circles with blue outlines represent simultaneous urines and scans.


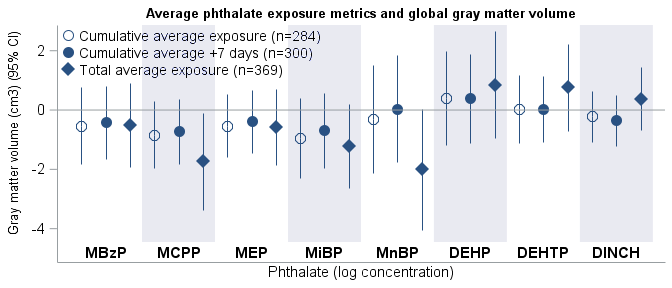

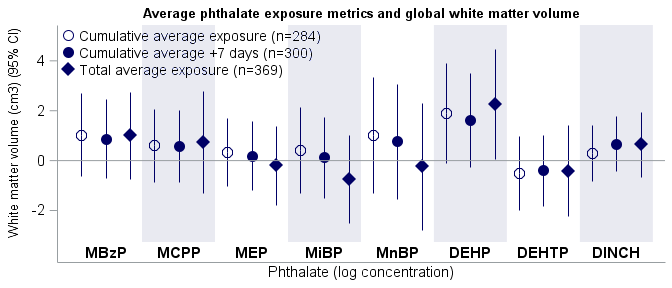

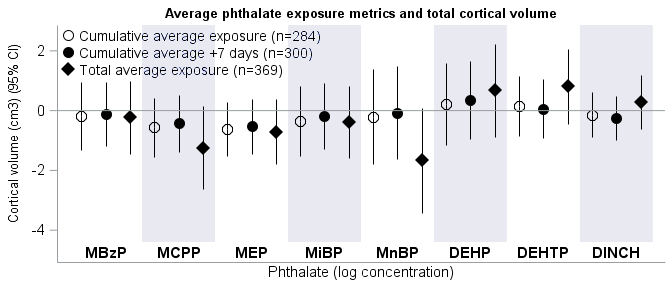


Supplemental Figure 2. Associations between different average phthalate exposure metrics (cumulative average, cumulative average plus seven days after scan, and total average across the study period) and global brain volumes (gray matter, white matter, total cortical).

Supplemental Table 1. Associations of **cumulative** average phthalate exposures with global gray and white matter volumes in the UNC BCP (n=284).

| Exposure | Brain volume | Overall (n=284) | Females (n=173) | Males (n=111) | Interaction p-value |
| --- | --- | --- | --- | --- | --- |
|  |  | β (95% CI) | β (95% CI) | β (95% CI) |  |
| MBzP | GMV | -0.5 (-1.8, 0.8) | -0.6 (-2.4, 1.1) | -0.3 (-2.5, 1.8) | 0.7 |
|  | WMV | 1.0 (-0.6, 2.7) | 0.1 (-2.3, 2.6) | 1.1 (-1.5, 3.8) | 0.5 |
| MCPP | GMV | -0.8 (-2.0, 0.3) | -0.7 (-2.0, 0.6) | -0.8 (-3.1, 1.4) | 0.9 |
|  | WMV | 0.6 (-0.9, 2.1) | 0.8 (-1.0, 2.5) | 0.3 (-2.6, 3.2) | 0.7 |
| MEP | GMV | -0.5 (-1.6, 0.5) | -0.4 (-1.5, 0.8) | -1.4 (-3.8, 1.0) | 0.6 |
|  | WMV | 0.4 (-1.0, 1.7) | -0.5 (-2.1, 1.0) | 1.3 (-1.8, 4.3) | 0.2 |
| MiBP | GMV | -1.0 (-2.3, 0.4) | 0.3 (-1.4, 2.1) | **-2.3 (-4.5, -0.1)** | 0.09 |
|  | WMV | 0.4 (-1.3, 2.2) | 0.3 (-2.0, 2.6) | 0.1 (-2.7, 2.9) | 0.9 |
| MnBP | GMV | -0.3 (-2.1, 1.5) | -0.1 (-2.2, 2.1) | -0.5 (-4.1, 3.0) | 1.0 |
|  | WMV | 1.0 (-1.3, 3.4) | 1.0 (-1.2, 3.3) | -0.5 (-4.9, 4.0) | 0.9 |
| ∑DEHP | GMV | 0.4 (-1.2, 2.0) | 1.3 (-0.5, 3.0) | -2.4 (-5.8, 1.0) | **0.04** |
|  | WMV | 1.9 (-0.1, 3.9) | 0.2 (-2.2, 2.6) | 2.4 (-1.9, 6.6) | 0.7 |
| ∑DEHTP | GMV | 0.0 (-1.1, 1.2) | 0.4 (-0.9, 1.8) | -0.6 (-2.8, 1.6) | 0.4 |
|  | WMV | -0.5 (-2.0, 1.0) | -0.5 (-2.2, 1.2) | -2.2 (-4.9, 0.5) | 0.1 |
| ∑DINCH | GMV | -0.2 (-1.1, 0.7) | -0.4 (-1.5, 0.6) | 0.4 (-1.2, 1.9) | 0.5 |
|  | WMV | 0.3 (-0.8, 1.4) | **1.5 (0.2, 2.8)** | -1.3 (-3.2, 0.7) | **0.01** |

GMV, gray matter volume (cm^3^); WMV, white matter volume (cm^3^); CI, confidence interval; MBzP, monobenzyl phthalate; MCPP, mono-3carboxypropyl phthalate; MEP, monoethyl phthalate; MiBP, monoisobutyl phthalate; MnBP, monobutyl phthalate; DEHP, di(2-ethylhexyl) phthalate; DEHTP, di-2-ethylhexyl terephthalate; DINCH, di(isononyl)cyclohexane-1,2-dicarboxylate; effect estimates (β coefficients) are per log-unit increase in SG-adjusted average urinary phthalate concentrations (log(ng/mL) for metabolites and log(µmol/L) for molar sums); models are adjusted for maternal age at delivery, child’s sex, child’s age (days) on the date of the MRI, and intracranial volume; sex-specific estimates are estimated from stratified models; interaction p-value is for product term between cumulative average phthalate exposure and child’s sex; boldface type indicates association with p-value < 0.05.

Supplemental Table 2. Associations of **cumulative** average phthalate exposures with cortical surface area, thickness, and volume in the UNC BCP (n=284).

| Phthalate | Cortical measure | Overall (n=284) | Females (n=173) | Males (n=111) | Interaction p-value |
| --- | --- | --- | --- | --- | --- |
|  |  | β (95% CI) | β (95% CI) | β (95% CI) |  |
| MBzP | CSA | 0.7 (-7.2, 8.5) | -2.7 (-13.3, 7.9) | 3.7 (-9.1, 16.6) | 0.7 |
|  | CT | 0.0004 (-0.01, 0.01) | 0.003 (-0.01, 0.01) | -0.002 (-0.01, 0.01) | 0.9 |
|  | CV | -0.2 (-1.3, 0.9) | -0.5 (-2.1, 1.1) | 0.4 (-1.5, 2.4) | 0.4 |
| MCPP | CSA | -0.5 (-7.3, 6.4) | 0.1 (-7.8, 8.1) | -2.2 (-15.7, 11.3) | 0.6 |
|  | CT | 0.00004 (-0.01, 0.01) | -0.001 (-0.01, 0.01) | 0.004 (-0.01, 0.02) | 0.3 |
|  | CV | -0.6 (-1.5, 0.4) | -0.5 (-1.7, 0.6) | -0.4 (-2.5, 1.6) | 1.0 |
| MEP | CSA | -0.9 (-7.2, 5.5) | -3.7 (-10.6, 3.3) | 4.1 (-10.4, 18.6) | 0.7 |
|  | CT | 0.001 (-0.005, 0.01) | 0.003 (-0.005, 0.01) | -0.003 (-0.02, 0.01) | 0.9 |
|  | CV | -0.6 (-1.5, 0.3) | -0.7 (-1.7, 0.2) | -0.8 (-3.0, 1.3) | 0.8 |
| MiBP | CSA | **9.9 (1.8, 18.0)** | **14.9 (4.6, 25.1)** | 4.9 (-8.7, 18.5) | 0.1 |
|  | CT | **-0.01 (-0.02, -0.01)** | **-0.01 (-0.03, -0.004)** | **-0.01 (-0.03, -0.001)** | 0.6 |
|  | CV | -0.4 (-1.5, 0.8) | 0.9 (-0.7, 2.4) | -1.2 (-3.2, 0.8) | 0.1 |
| MnBP | CSA | 7.4 (-3.5, 18.3) | 9.4 (-3.6, 22.4) | 0.8 (-20.3, 22.0) | 0.3 |
|  | CT | -0.01 (-0.02, 0.002) | -0.01 (-0.02, 0.01) | -0.01 (-0.03, 0.01) | 0.6 |
|  | CV | -0.2 (-1.8, 1.4) | 1.0 (-0.5, 2.5) | -0.2 (-3.4, 3.0) | 0.9 |
| ∑DEHP | CSA | 8.2 (-1.2, 17.6) | 10.2 (-0.3, 20.6) | -1.2 (-21.8, 19.5) | 0.5 |
|  | CT | -0.004 (-0.01, 0.01) | -0.001 (-0.01, 0.01) | -0.01 (-0.03, 0.01) | 0.2 |
|  | CV | 0.2 (-1.1, 1.6) | 1.1 (-0.5, 2.6) | -1.7 (-4.8, 1.4) | 0.2 |
| ∑DEHTP | CSA | 2.4 (-4.5, 9.3) | 5.4 (-2.7, 13.6) | -4.4 (-17.6, 8.9) | 0.3 |
|  | CT | -0.001 (-0.01, 0.01) | -0.001 (-0.0101, 0.01) | -0.00004 (-0.01, 0.01) | 0.9 |
|  | CV | 0.1 (-0.9, 1.2) | 0.5 (-0.6, 1.7) | -0.6 (-2.6, 1.4) | 0.3 |
| ∑DINCH | CSA | -2.2 (-7.4, 3.0) | -1.3 (-7.7, 5.1) | -3.2 (-12.4, 6.0) | 0.8 |
|  | CT | 0.002 (-0.003, 0.01) | 0.001 (-0.01, 0.01) | 0.004 (-0.005, 0.01) | 0.8 |
|  | CV | -0.1 (-0.9, 0.6) | **1.1 (0.4, 1.8)** | 0.1 (-1.3, 1.5) | 0.6 |

CSA, cortical surface area (cm^2^); CT, cortical thickness (mm); CV, cortical volume (cm^3^); CI, confidence interval; MBzP, monobenzyl phthalate; MCPP, mono-3carboxypropyl phthalate; MEP, monoethyl phthalate; MiBP, monoisobutyl phthalate; MnBP, monobutyl phthalate; DEHP, di(2-ethylhexyl) phthalate; DEHTP, di-2-ethylhexyl terephthalate; DINCH, di(isononyl)cyclohexane-1,2-dicarboxylate; effect estimates (β coefficients) are per log-unit increase in SG-adjusted average urinary phthalate concentrations (log(ng/mL) for metabolites and log(µmol/L) for molar sums); models are adjusted for maternal age at delivery, child’s sex, child’s age (days) on the date of the MRI, and intracranial volume; sex-specific estimates are estimated from stratified models; interaction p-value is for product term between cumulative average phthalate exposure and child’s sex; boldface type indicates association with p-value < 0.05.

Supplemental Table 3. Age-stratified associations of total average phthalate exposures with gray and white matter volume, and cortical surface area, thickness, and volume dichotomized at age 2 years old among children in the UNC BCP (n=225).

| Phthalate | MRI measure | Age ≤ 2y (n=225) | Age > 2y (n=156) | Interaction  p-value |
| --- | --- | --- | --- | --- |
|  |  | β (95% CI) | β (95% CI) |  |
| MBzP | GMV | -0.9 (-3.0, 1.3) | -0.1 (-2.4, 2.2) | 0.6 |
|  | WMV | 0.3 (-2.6, 3.1) | 2.3 (-0.4, 5.0) | 0.3 |
|  | CSA | -2.6 (-15.7, 10.5) | 4.1 (-10.2, 18.4) | 0.2 |
|  | CT | 0.00 (-0.012, 0.012) | -0.006 (-0.019, 0.007) | 0.2 |
|  | CV | -0.1 (-2.2, 1.9) | -0.3 (-2.2, 1.6) | 0.8 |
| MCPP | GMV | **-2.7 (-4.7, -0.6)** | -0.8 (-4.6, 2.9) | 0.5 |
|  | WMV | -0.4 (-3.3, 2.4) | 1.7 (-2.6, 6.1) | 0.5 |
|  | CSA | -7.3 (-20.1, 5.6) | 9.0 (-13.9, 31.9) | 0.2 |
|  | CT | -0.002 (-0.01, 0.01) | -0.01 (-0.03, 0.01) | 0.7 |
|  | CV | **-2.3 (-4.3, -0.4)** | -0.2 (-3.2, 2.8) | 0.3 |
| MEP | GMV | -0.3 (-2.1, 1.5) | -2.0 (-4.4, 0.4) | 0.2 |
|  | WMV | -0.8 (-3.2, 1.7) | -1.6 (-4.5, 1.2) | 0.5 |
|  | CSA | -4.8 (-15.9, 6.4) | -6.2 (-21.1, 8.8) | 0.8 |
|  | CT | 0.01 (-0.004, 0.02) | -0.005 (-0.02, 0.01) | 0.1 |
|  | CV | -0.6 (-2.3, 1.2) | -**2.2 (-4.1, -0.3)** | 0.1 |
| MiBP | GMV | **-2.3 (-4.1, -0.4)** | -0.1 (-3.0, 2.9) | 0.2 |
|  | WMV | -1.0 (-3.6, 1.6) | -1.0 (-4.4, 2.5) | 0.9 |
|  | CSA | 2.9 (-9.0, 14.7) | 13.2 (-4.7, 31.2) | 0.3 |
|  | CT | **-0.01 (-0.02, -0.0001)** | -0.01 (-0.03, 0.01) | 0.9 |
|  | CV | -1.4 (-3.2, 0.4) | 0.4 (-1.9, 2.8) | 0.3 |
| MnBP | GMV | -1.9 (-4.8, 0.9) | -2.8 (-6.7, 1.1) | 0.5 |
|  | WMV | -2.5 (-6.3, 1.3) | 1.8 (-2.8, 6.4) | 0.3 |
|  | CSA | -2.1 (-19.7, 15.4) | 0.9 (-23.2, 25.0) | 0.7 |
|  | CT | -0.01 (-0.02, 0.01) | -0.01 (-0.03, 0.01) | 0.6 |
|  | CV | -2.0 (-4.7, 0.8) | -2.2 (-5.3, 0.9) | 0.6 |
| ∑DEHP | GMV | 0.0 (-2.8, 2.8) | 0.8 (-2.3, 3.8) | 0.9 |
|  | WMV | -0.9 (-4.7, 2.9) | **3.9 (0.5, 7.4)** | 0.08 |
|  | CSA | 8.9 (-8.5, 26.4) | 10.4 (-8.4, 29.1) | 0.9 |
|  | CT | -0.01 (-0.02, 0.01) | -0.005 (-0.02, 0.01) | 1.0 |
|  | CV | -0.5 (-3.2, 2.3) | 0.8 (-1.6, 3.3) | 0.8 |
| ∑DEHTP | GMV | -0.7 (-3.0, 1.5) | 2.2 (-0.2, 4.7) | 0.1 |
|  | WMV | 0.3 (-2.7, 3.4) | -1.6 (-4.5, 1.3) | 0.3 |
|  | CSA | 2.1 (-11.9, 16.1) | 4.3 (-11.0, 19.6) | 0.5 |
|  | CT | -0.01 (-0.02, 0.004) | 0.005 (-0.01, 0.02) | **0.03** |
|  | CV | -0.4 (-2.6, 1.8) | 1.8 (-0.1, 3.8) | 0.2 |
| ∑DINCH | GMV | -0.5 (-1.9, 1.0) | 1.4 (-0.5, 3.2) | 0.09 |
|  | WMV | 0.7 (-1.3, 2.6) | -1.3 (-3.5, 0.9) | **0.04** |
|  | CSA | -2.1 (-10.9, 6.8) | 3.0 (-8.7, 14.7) | 0.5 |
|  | CT | 0.002 (-0.01, 0.01) | 0.001 (-0.01, 0.01) | 1.0 |
|  | CV | -0.5 (-1.9, 0.9) | 1.1 (-0.4, 2.6) | 0.1 |

GMV, gray matter volume (cm^3^); WMV, white matter volume (cm^3^); CSA, cortical surface area (cm^2^); CT, cortical thickness (mm); CV, cortical volume (cm^3^); CI, confidence interval; MBzP, monobenzyl phthalate; MCPP, mono-3carboxypropyl phthalate; MEP, monoethyl phthalate; MiBP, monoisobutyl phthalate; MnBP, monobutyl phthalate; DEHP, di(2-ethylhexyl) phthalate; DEHTP, di-2-ethylhexyl terephthalate; DINCH, di(isononyl)cyclohexane-1,2-dicarboxylate; effect estimates (β coefficients) are per log-unit increase in SG-adjusted average urinary phthalate concentrations (log(ng/mL) for metabolites and log(µmol/L) for molar sums); models are adjusted for maternal age at delivery, child’s sex, child’s age (days) on the date of the MRI, and intracranial volume; age group-specific estimates are estimated from stratified models; interaction p-value is for product term between total average phthalate exposure and child’s age (dichotomized at 2 years of age); boldface type indicates association with p-value < 0.05.
